# Imagem Cardiovascular e Procedimentos Intervencionistas em Pacientes com Infecção pelo Novo Coronavírus

**DOI:** 10.36660/abc.20200370

**Published:** 2020-07-28

**Authors:** Isabela Bispo Santos da Silva Costa, Carlos Eduardo Rochitte, Carlos M. Campos, Silvio Henrique Barberato, Gláucia Maria Moraes de Oliveira, Marcelo Antônio Cartaxo Queiroga Lopes, Cesar Higa Nomura, Alexandre A. Abizaid, Giovanni Cerri, Roberto Kalil, Ludhmila Abrahão Hajjar

**Affiliations:** 1 Universidade de São Paulo Instituto do Câncer do Estado de São Paulo São PauloSP Brasil Universidade de São Paulo Instituto do Câncer do Estado de São Paulo , São Paulo , SP - Brasil; 2 Universidade de São Paulo Instituto do Coração São PauloSP Brasil Universidade de São Paulo Instituto do Coração , São Paulo , SP - Brasil; 3 Hospital do Coração São PauloSP Brasil Hospital do Coração , São Paulo , SP - Brasil; 4 Hospital Pró-Cardíaco Rio de JaneiroRJ Brasil Hospital Pró-Cardíaco , Rio de Janeiro , RJ - Brasil; 5 Universidade de São Paulo Instituto do Coração São PauloSP Brasil Universidade de São Paulo Instituto do Coração - Hemodinâmica e Cardiologia Intervencionista, São Paulo , SP - Brasil; 6 Hospital Israelita Albert Einstein São PauloSP Brasil Hospital Israelita Albert Einstein - Hemodinâmica e Cardiologia Intervencionista, São Paulo , SP - Brasil; 7 CardioEco CuritibaPR Brasil CardioEco -Centro de Diagnóstico Cardiovascular, Curitiba , PR - Brasil; 8 Quanta Diagnóstico CuritibaPR Brasil Quanta Diagnóstico – Ecocardiografia, Curitiba , PR - Brasil; 9 Universidade Federal do Rio de Janeiro Rio de JaneiroRJ Brasil Universidade Federal do Rio de Janeiro – Cardiologia, Rio de Janeiro , RJ - Brasil; 10 Hospital Alberto Urquiza Wanderley João PessoaPB Brasil Hospital Alberto Urquiza Wanderley - Hemodinâmica e Cardiologia Intervencionista, João Pessoa , PB - Brasil; 11 Hospital Metropolitano Dom José Maria Pires João PessoaPB Brasil Hospital Metropolitano Dom José Maria Pires , João Pessoa , PB - Brasil; 12 Sociedade Brasileira de Cardiologia Rio de JaneiroRJ Brasil Sociedade Brasileira de Cardiologia , Rio de Janeiro , RJ - Brasil; 13 Hospital Sírio Libanês São PauloSP Brasil Hospital Sírio Libanês , São Paulo , SP - Brasil

**Keywords:** Coronavírus, COVID-19, Pandemia, Doenças Infecciosas Emergentes, Doenças Cardiovasculares/prevenção e controle, Diagnóstico por Imagem, Orientação, Exames Médicos/métodos, Técnicas e Procedimentos Diagnósticos

## Abstract

A pandemia da doença causada pelo novo coronavírus (COVID-19) trouxe grandes desafios para o sistema de saúde devido ao aumento exponencial do número de pacientes acometidos. A racionalização de recursos e a indicação correta e criteriosa de exames de imagem e procedimentos intervencionistas tornaram-se necessárias, priorizando a segurança do paciente, do ambiente e dos profissionais da saúde. Esta revisão visa auxiliar e orientar os profissionais envolvidos na realização desses exames e procedimentos a fazê-los de forma eficaz e segura.

## 1. Introdução

A COVID-19, doença causada pelo novo coronavírus (SARS-CoV-2), é um grande desafio atual para a sociedade. Sua rápida disseminação fez com que se tornasse uma pandemia, tendo atingido em poucos meses 185 países, resultando em 4.995.127 infectados (20 de maio) e 328.079 mortes. ^1^ As estatísticas brasileiras demonstram números em ascensão, apesar da subnotificação pela indisponibilidade de testes, tendo-se registrado até o momento 291.579 infectados e 18.859 mortes. ^2^

A estratégia mais efetiva para controlar a transmissão da doença é o isolamento, com medidas como quarentena e distanciamento social. ^3^ Os hospitais, clínicas e consultórios têm seguido recomendações de sociedades médicas nacionais e internacionais no sentido de proteger ao máximo os pacientes sem COVID-19 do risco de infecção e, ao mesmo tempo, propiciar manejo adequado àqueles com COVID-19. ^4 , 5^ Nessa premissa, procedimentos considerados eletivos deveriam ser reagendados para momento oportuno. ^6^

O manejo adequado dos pacientes infectados compreende uma série de medidas que envolve a interação entre diversos setores hospitalares e uma equipe multidisciplinar treinada. A grande maioria dos infectados que evoluem com a forma mais grave da COVID-19 apresenta comorbidades, sendo frequentes as doenças cardiovasculares. ^7 - 9^ Além disso, complicações cardiovasculares da COVID-19 ocorrem entre 7% e 40% dos casos, manifestando-se como injúria miocárdica, trombose, disfunção ventricular, miocardite, arritmias e choque. ^10 - 12^ O desenvolvimento dessas complicações tem implicações prognósticas importantes, com elevada taxa de mortalidade. ^11^

Para o diagnóstico e o seguimento dos pacientes com essas complicações, frequentemente são necessários exames de imagem, como eletrocardiograma, ecocardiograma transtorácico (ETT), tomografia computadorizada (TC) e, em alguns pacientes, ressonância magnética cardíaca (RMC) e angiotomografia de artérias coronárias (ATAC). Esses exames não devem ser realizados de rotina em todos os infectados, sendo sua indicação preferencialmente pautada no benefício adicionado ao cuidado do paciente e levando-se em consideração a segurança da equipe que conduzirá o exame. O uso racional, responsável e criterioso dos recursos faz com que o clínico desempenhe papel importante na identificação do paciente que necessita do exame, na seleção adequada do exame e na interpretação correta dos achados.

Dessa forma, esta revisão tem como objetivos: a) auxiliar na indicação dos exames cardiovasculares e procedimentos intervencionistas e na sua implementação na prática clínica para pacientes com suspeita ou confirmação de COVID-19; b) orientar os médicos que realizarão os exames e os procedimentos a fazê-los de forma segura, evitando a contaminação do ambiente e dos profissionais de saúde.

## 2. Abordagem do Paciente Com Suspeita ou Confirmação de Covid-19

A abordagem do paciente com suspeita ou confirmação de COVID-19 deve se iniciar pela caracterização adequada dos sinais e sintomas apresentados. Os pacientes com sintomas gripais leves (tosse, febre, dor de garganta) podem ser acompanhados em unidades de baixa complexidade ou em regime domiciliar. Aqueles com sintomas que denotem gravidade (saturação de oxigênio <94% em ar ambiente, desconforto respiratório, taquipneia, hipotensão, insuficiência respiratória aguda) devem ser encaminhados para centros especializados. No contato inicial, deve ser fornecida máscara cirúrgica para a pessoa sintomática, que deve ser direcionada para sala específica, visando isolamento respiratório, e deve receber material para higiene das mãos, evitando o contágio do ambiente e de outras pessoas. ^4^

A identificação dos pacientes de risco deve incluir a avaliação da presença de comorbidades clínicas que sabidamente estão relacionadas à evolução mais grave da doença. ^7 , 13^ Pacientes com hipertensão (HAS), doença cardiovascular crônica, diabetes mellitus (DM), doença pulmonar obstrutiva crônica (DPOC) ou doença renal crônica, ou ainda pacientes imunossuprimidos ou idosos, são mais suscetíveis a desenvolverem complicações, devendo ser considerados grupo de risco. ^4^

Pacientes com sintomas graves e/ou do grupo de risco são mais propensos a complicações cardiovasculares relacionadas à COVID-19. ^8 , 9^ Zhou et al., ^9^ em uma coorte com 191 pacientes, observaram prevalência elevada de HAS (30%), DM (19%), doença arterial coronariana (DAC - 8%) e DPOC (3%). ^9^ Dos 54 pacientes (28%) que evoluíram a óbito, 67% apresentavam alguma comorbidade, 48% apresentavam HAS, 31% apresentavam DM e 24% apresentavam DAC. Idade avançada foi preditor independente de mortalidade. ^9^

Outro marcador importante de gravidade desses doentes é a presença de níveis séricos aumentados de troponina, NT-proBNP e dímero-D. Os pacientes com elevação de troponina evoluíram com formas mais graves da COVID-19, com incidência aumentada de síndrome do desconforto respiratório agudo (SDRA) e morte. ^11^ A elevação de troponina é acompanhada de elevação de marcadores inflamatórios, trombóticos e de disfunção cardíaca, e os pacientes com essa característica têm maior chance de evoluir para quadros de falência cardíaca aguda e choque. ^8 , 9 , 11^

À admissão, os pacientes que apresentarem critérios clínicos ou laboratoriais sugestivos de maior gravidade devem ter sua função cardiovascular estudada por meio de avaliação clínica, dosagem de biomarcadores e realização de exames de imagem, conforme descrição abaixo. ^12 , 14^ Os exames de imagem e os procedimentos intervencionistas mais comumente realizados são descritos a seguir.

## 3. Ecocardiografia

A ecocardiografia tem papel estabelecido no diagnóstico, na avaliação prognóstica e na orientação terapêutica em diversas doenças cardiovasculares. Entretanto, como implica contato próximo entre o examinador e o paciente, traz alto risco de infecção por SARS-CoV-2. A pandemia tornou urgente a reorganização dos laboratórios de ecocardiografia para minimizar a exposição à COVID-19 e assegurar a proteção de pacientes, médicos e equipe de trabalho. ^5^ Dessa forma, o Departamento de Imagem Cardiovascular / Sociedade Brasileira de Cardiologia lançou um documento para auxiliar os profissionais durante esta pandemia. ^5^

De forma geral, a ecocardiografia não deve ser realizada de rotina durante a pandemia, especialmente em pacientes com COVID-19 confirmada. Por outro lado, os profissionais da ecocardiografia continuarão sendo expostos em determinados cenários clínicos nos quais o ecocardiograma pode ser decisivo no diagnóstico diferencial e no manejo clínico dos pacientes mais graves. Sabe-se que a COVID-19 pode gerar manifestações cardiovasculares graves e que a doença cardiovascular prévia é comum nos pacientes com COVID-19, estando associada com pior prognóstico. ^9 , 14 , 15^

### 3.1. Precauções Gerais

Todo e qualquer atendimento durante a pandemia deve seguir as seguintes recomendações para minimizar os riscos de exposição do profissional de saúde e dos pacientes à COVID-19: (a) definir se o exame é considerado essencial naquele momento; (b) realizar triagem prévia quanto ao risco de contaminação; (c) respeitar as normas gerais de higienização das mãos e restrição de contato; e (d) utilizar de forma rigorosa e racional o equipamento de proteção individual (EPI) adequado, conforme o tipo do exame e o risco de contaminação. ^5^

Nos casos de baixo risco de COVID-19 (áreas de baixo risco e teste negativo para o vírus), para os pacientes assintomáticos que serão submetidos ao ETT, preconiza-se a criteriosa lavagem das mãos e a utilização de luvas e máscara cirúrgica para o ecocardiografista. É recomendável que o paciente de baixo risco use também máscara durante o atendimento. Nos casos de moderado a alto risco (pacientes sintomáticos com COVID-19 suspeita ou confirmada), as normas de segurança incluem higienização das mãos, uso de luvas, máscara cirúrgica (ou N95, caso disponível), avental, gorro e proteção ocular (óculos ou escudo facial) para o examinador. A máscara é obrigatória para o paciente nesse caso. Na eventualidade da realização do ecocardiograma transesofágico (ETE), é obrigatória a adição da máscara N95 ou similar ao aparato anterior, configurando precaução aérea, uma vez que o procedimento é capaz de gerar aerossóis contendo grande quantidade de vírus. É recomendável também utilizar proteção para os pés (botas ou propés) e capa protetora para o transdutor, se possível. Em pacientes com COVID-19 suspeita ou confirmada e sob ventilação mecânica não invasiva ou invasiva, a precaução aérea também deve ser adotada para a execução do ETT. ^16^ Em pacientes internados, a preferência é fazer os ecocardiogramas necessários à beira do leito, com as medidas de proteção adequadas e com o menor número possível de indivíduos no aposento. Devem ser feitas a limpeza e a desinfecção apropriadas das máquinas e transdutores logo após o uso, de acordo com as especificações de cada fabricante. Integrantes do laboratório de ecocardiografia acima de 60 anos de idade, portadores de doenças crônicas, imunodeprimidos e gestantes devem idealmente ser afastados. ^5 , 17^

### 3.2. Indicações de Ecocardiografia em Pacientes de Baixo Risco para Covid-19

Durante a pandemia, as solicitações de ecocardiografia em indivíduos com baixo risco de COVID-19 devem ser baseadas nas indicações de uso apropriado e devem ser realizadas somente se a informação gerada pelo exame for essencial para o manejo do caso. ^5^ Todos os exames considerados eletivos devem ser adiados para quando as operações voltarem ao normal, incluindo ETT, ETE, ecocardiograma sob estresse (ESE) e ecocardiograma fetal (EF). A urgência da realização de um ecocardiograma em paciente ambulatorial deve ser avaliada caso a caso, mas sugere-se ser urgente aquele exame cujo resultado possa prevenir um evento adverso ou uma internação hospitalar em espaço de tempo de 2 a 4 semanas. ^17^ Como sugestões de recomendação de ecocardiografia nesses cenários, incluem-se: suspeita de nova cardiomiopatia francamente sintomática [classe funcional III/IV da *New York Heart Association* (NYHA)]; piora de insuficiência cardíaca preexistente com sintomas graves (síncope, dor torácica, classe funcional III/IV da NYHA); terapia do câncer em uso de medicação cardiotóxica com suspeita de insuficiência cardíaca ou queda prévia da fração de ejeção; suspeita de estenose aórtica grave sintomática; alta probabilidade pré-teste de endocardite infecciosa em portador de prótese valvar com sintomas agudos. ^17^ A ecocardiografia de rotina para seguimento de pacientes com sintomas não graves ou daqueles não elegíveis para terapia clínica, cirúrgica ou invasiva urgente deve ser adiada ou cancelada. Em pacientes internados, as indicações de ecocardiograma de urgência em geral são as mesmas que antes da pandemia.

### 3.3. Indicações de Ecocardiografia em Pacientes com Covid-19 Suspeita ou Confirmada

A ecocardiografia permanece um método crucial de imagem durante o surto de coronavírus e a consideração de “em quem”, “como” e “onde” empregá-la é fundamental para diminuir os riscos de contaminação e, ao mesmo tempo, garantir cuidado médico de alta qualidade. Alguns autores advogam a realização do ETT em todos os pacientes com COVID-19 complicada (alterações eletrocardiográficas, aumento de troponinas, sintomas moderados a graves requerendo internação), ^12 , 18^ especialmente se houver doença cardiovascular prévia. Embora não existam indicações formais fundamentadas em evidências científicas sólidas, enfatiza-se a importância de avaliar a função cardíaca frente à potencial coincidência de doença cardiovascular prévia e aguda nos pacientes com COVID-19 grave.

Zhou et al., ^9^ relataram que insuficiência cardíaca esteve presente em 23% dos pacientes com COVID-19 e se associou com maior mortalidade (51,9% versus 11,7%). ^9^ Não está claro se a taxa de insuficiência cardíaca se deveu a exacerbação de disfunção ventricular prévia, nova cardiomiopatia ou ambas. É plausível que pacientes com disfunção ventricular prévia possam desenvolver profunda descompensação da insuficiência cardíaca no contexto da COVID-19 grave, acompanhada de hipotensão e/ou choque cardiogênico. Aventam-se várias possibilidades para a lesão miocárdica aguda, como ação viral direta (miocardite), injúria hipóxica, efeito tóxico pela “tempestade” de citocinas, vasoespasmo, trombose, *stunning* miocárdico por cardiomiopatia de estresse ou instabilidade hemodinâmica. ^19 - 21^ Muito se tem discutido no meio científico a possibilidade de o SARS-CoV-2 causar miocardite. Em uma análise retrospectiva de 68 mortes ocorridas em uma série de 150 pacientes com COVID-19, 53% foram atribuídas à insuficiência respiratória, 7% à miocardite com choque circulatório, 33% à combinação de ambas as anteriores, restando 5% de causa desconhecida. ^15^ Os autores empregaram dados clínicos para diagnosticar miocardite fulminante, sem comprovação por biópsia. Da mesma forma, relatos de miocardite fulminante foram publicados em pacientes com ou sem febre que tiveram dor torácica, elevação do segmento ST sem obstrução coronariana e disfunção ventricular grave, respondendo à terapia salvadora com corticoide e imunoglobulinas. ^22 , 23^ Embora nesses dois relatos a RMC tenha demonstrado achados compatíveis com miocardite, não houve constatação histológica comprobatória. ^22 , 23^

No diagnóstico diferencial com miocardite e cardiomiopatia de estresse, é obviamente necessária a inclusão das síndromes coronarianas agudas, que também têm sido descritas em pacientes com COVID-19. ^24 , 25^ Acredita-se que a intensa resposta inflamatória e as alterações hemodinâmicas associadas com a doença grave possam conferir maior risco de ruptura de placas ateroscleróticas e/ou fenômenos tromboembólicos em pacientes suscetíveis. ^14^ Pelo acima exposto, mesmo em pacientes sem febre ou tosse, que cursam com manifestação clínica tipicamente cardíaca, a COVID-19 deve entrar no diagnóstico diferencial durante a pandemia e a ecocardiografia pode auxiliar no julgamento clínico.

Arritmias cardíacas são manifestações comuns em pacientes com COVID-19 hospitalizados, sendo descritas em 16,7% dos casos em coorte chinesa com 138 pacientes. ^7^ A ecocardiografia pode ser útil em alguns casos, especialmente para arritmias ventriculares malignas, ao diagnosticar disfunção ventricular esquerda ou cardiopatia estrutural preexistente.

No contexto da pneumopatia grave e SDRA, torna-se importante avaliar hipertensão pulmonar e disfunção do ventrículo direito associada. Derrame pericárdico tem sido relatado como achado de exame em associação com miocardite (miopericardite), em geral sem repercussão hemodinâmica significativa. ^22 , 23^

Configuram-se assim alguns cenários clínicos em que a indicação de ecocardiografia em pacientes com COVID-19 parece ser justificável: ^12 , 17 , 18 , 26^

- Suspeita de insuficiência cardíaca
- Cardiomegalia na radiografia de tórax
- Arritmias clinicamente significativas
- Dor torácica com alterações eletrocardiográficas e/ou elevação de troponinas
- Instabilidade hemodinâmica e/ou choque
- Suspeita de hipertensão pulmonar e/ou disfunção ventricular direita

Recomenda-se a utilização de ecocardiograma à beira do leito no paciente grave internado em terapia intensiva na admissão e durante a evolução, devendo ser preferencialmente pelo método *point-of-care* . ^5 , 12^

### 3.4. Protocolos Especiais Durante a Pandemia

**3.4.1. Ecocardiograma transtorácico:** deve-se diminuir o tempo de realização dos exames, direcionando para a suspeita diagnóstica em questão. Como a duração prolongada do exame aumenta o risco de contaminação, diversas instituições têm recomendado o ecocardiograma focado em detrimento do ETT completo. ^5 , 17 , 26^ Por outro lado, deve-se evitar a repetição desnecessária dos exames e, de acordo com a complexidade do caso, o ETT completo pode ser requerido para atender à demanda clínica. As imagens devem ser armazenadas para realização de medidas *off-line* e a monitorização por eletrocardiograma pode ser dispensada. O ideal é ter um ecocardiógrafo exclusivo dedicado para uso em pacientes com COVID-19 e que deve permanecer nas áreas contaminadas. Medidas de proteção adicionais podem ser empregadas, como cobrir o aparelho com papel filme e/ou a interposição de barreira de acrílico (ou plástico) entre o examinador e o paciente. Os aparelhos portáteis ou ultrassom de bolso apresentam vantagens pela facilidade de cobrir, transportar e desinfetar, porém têm recursos diagnósticos limitados ( *point-of-care* ). O contraste ecocardiográfico pode ser útil em alguns casos e seu emprego deve ser antecipado para evitar saídas e entradas adicionais do aposento para obtê-lo, ^26^ fazendo-se a ressalva de não usar em pacientes críticos com instabilidade circulatória e grave comprometimento pulmonar.

**3.4.2. Ecocardiograma focado ( *point-of-care* ):** pode desempenhar importante papel no cuidado dos pacientes críticos em tempos de COVID-19. Não equivale ao ETT completo, porém é capaz de confirmar ou excluir um diagnóstico específico, facilitando decisões terapêuticas. ^27^ Pode ser realizado por médicos adequadamente treinados que já estão envolvidos no cuidado direto ao paciente na unidade de terapia intensiva, dessa forma contribuindo para diminuir a exposição do profissional da ecocardiografia. De preferência, aparelhos portáteis ou ultrassom de bolso devem ser empregados para facilitar o acesso ao leito e posterior desinfecção.

**3.4.3. Ecocardiograma transesofágico:** preocupação especial existe com o ETE, pois o risco de contaminação do equipamento e dos profissionais de saúde por gotículas e aerossóis é muito alto. Assim, o valor incremental do ETE sobre o ETT deve ser cuidadosamente analisado, evitando-se o ETE na maioria dos casos. ^16^ Sempre que possível, outras alternativas devem ser consideradas, tais como repetir o ETT ou empregar outro método de imagem com menor contato entre o examinador e o paciente, como a TC e a RMC. Para se realizar o ETE de urgência em pacientes internados, usar EPI completo para precaução aérea e, se possível, capa protetora para o transdutor.

**3.4.4. Ecocardiograma sob estresse:** O ecocardiograma sob estresse físico pode aumentar o risco de contaminação por gotículas e deve ser adiado (em pacientes com baixo risco de COVID-19) ou não realizado (pacientes com COVID-19 suspeita ou confirmada). Em pacientes de baixo risco para COVID-19 que enfrentam situações em que a indicação é apropriada e o adiamento não é possível ou não recomendável (por exemplo, pré-operatório de cirurgia em paciente com câncer e probabilidade pré-teste alta de DAC obstrutiva), o ecocardiograma sob estresse farmacológico deve ser preferido. Uma alternativa para a investigação de casos selecionados de DAC crônica durante a pandemia é priorizar a realização de ATAC.

**3.4.5. Ecocardiograma fetal:** as indicações apropriadas de EF permanecem as mesmas durante a pandemia, ou seja, gestações com alto risco de cardiopatia fetal. ^16^ Recomenda-se que os exames sejam feitos fora do ambiente hospitalar. Realização de rotina do EF em mães com COVID-19 suspeita ou confirmada não encontra respaldo no momento.

**3.4.6. Ultrassom pulmonar:** consiste em uma alternativa ágil para avaliar o grau de envolvimento pulmonar e acompanhar o resultado de intervenções terapêuticas à beira do leito. Na COVID-19, foram descritas alterações da linha pleural (espessamento, irregularidade e perda de continuidade), aparecimento e progressão das linhas B (focais, multifocais, confluentes) e consolidação pulmonar (área patológica desprovida de ar, frequentemente subpleural). ^28^ Embora essas anormalidades sejam inespecíficas e encontradas em outros tipos de pneumonia, têm valor no seguimento evolutivo da pneumonia da COVID-19. O derrame pleural parece ser infrequente e o aparecimento de linhas A é observado na fase de recuperação pulmonar.

## 4. Radiografia de Tórax, Tomografia e Ressonância Cardiovascular

Os exames de imagem pulmonar e cardiovascular têm papel importante no diagnóstico correto de complicações resultantes da COVID-19. A radiografia de tórax é o exame mais utilizado nesses pacientes, mas por vezes é necessária a realização de TC. ^16^ A RMC pode ser necessária em pacientes com suspeita de miocardite e/ou síndrome de Takotsubo. ^16^ No entanto, algumas considerações sobre a realização da TC e da RMC antes da indicação do exame devem ser feitas. Esses exames apresentam risco potencial significativo de contaminação de pacientes e profissionais, principalmente os riscos ligados ao transporte, mas também à contaminação direta durante o exame. A RMC e a TC só devem ser realizadas se as informações esperadas forem auxiliar no manejo clínico do paciente. Essas modalidades devem ser indicadas em pacientes estáveis, cujo risco ao transporte seja mínimo, além de em ambiente seguro, com respeito às regras de segurança local e com os profissionais envolvidos no transporte e na aquisição das imagens usando EPI. ^16^

### 4.1. Radiografia de Tórax

**Figura 1:**
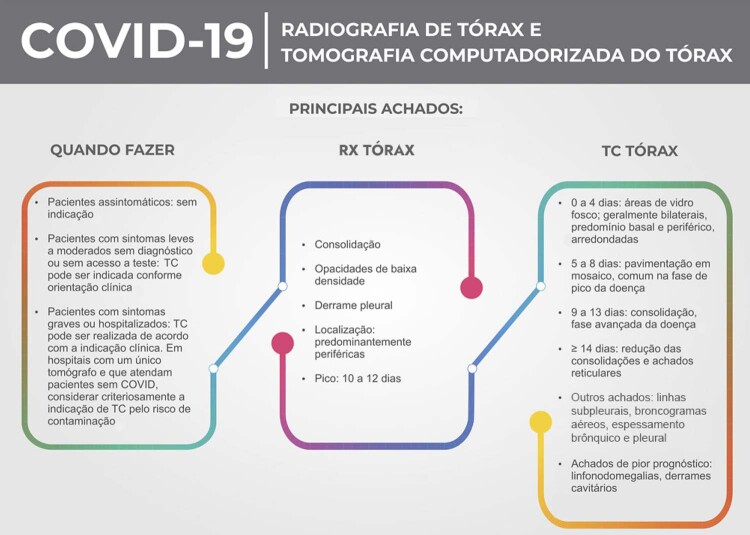
– *Radiografia de tórax e tomografia computadorizada de tórax na COVID-19. RX, radiografia; TC, tomografia computadorizada.*

A radiografia é normalmente o primeiro exame de imagem a ser realizado nos pacientes com COVID-19 pelo baixo custo e facilidade de acesso, especialmente nos hospitalizados que não podem ser transportados de forma segura. ^29^ Esse exame apresenta baixa sensibilidade e as alterações não são específicas para COVID-19, sendo comumente encontradas nas síndromes gripais. Observam-se consolidações (47%), opacidades de baixa densidade (33%) e derrame pleural (3%). ^30^ As alterações têm localização predominantemente periférica, ocorrendo com maior frequência em 10 a 12 dias. ^30^ Na Figura 1 , encontram-se resumidos os principais achados na radiografia de tórax.

### 4.2. Tomografia Computadorizada do Tórax

A TC é uma ferramenta auxiliar no diagnóstico, sendo a confirmação da COVID-19 baseada nos exames sorológicos ou de RT-PCR (do inglês, *reverse transcriptase polymerase chain reaction* ) viral. O uso da TC como *screening* deve ser desencorajado. ^29^ Os pacientes assintomáticos ou com sintomas leves não devem ser submetidos a TC. Seu uso em pacientes pouco sintomáticos e sem acesso à realização dos testes de RT-PCR ou sorológicos é incerto. Nos sintomáticos graves, internados, com condições seguras para transporte, que envolvem o uso de máscara pelo paciente, a TC deve ser considerada quando há suspeita de complicações (tromboembolismo pulmonar, derrame pleural e sobreposição de infecção bacteriana). ^29^ A Figura 1 sumariza os achados e as recomendações para a realização da TC.

O protocolo recomenda o uso de doses baixas de radiação, preferencialmente sem a administração de contraste, que deve ser reservado para indicações específicas, como para excluir tromboembolismo pulmonar. ^29^ A TC pode ser normal nos primeiros dias de evolução, o que não exclui o diagnóstico. A sensibilidade e a especificidade relatadas da TC para COVID-19 variam muito (60% a 98% e 25% a 53%, respectivamente), provavelmente devido à natureza retrospectiva dos estudos publicados, incluindo a falta de critérios rigorosos para diagnóstico de imagens e diferenças de procedimento para confirmar a infecção. ^31^

Os achados da TC variam de acordo com o tempo da doença. Na fase inicial, 57-98% dos pacientes vão apresentar opacidades em vidro fosco, geralmente bilaterais, periféricas e arredondadas. ^32^ De 5% a 36% dos pacientes vão apresentar pavimentação em mosaico na fase de pico da doença (5 a 8 dias após o início dos sintomas). As consolidações estão presentes em 2% a 64% dos pacientes, comumente encontradas nos idosos e com a forma grave da doença. Na fase mais tardia, observam-se a presença de padrão reticular em 48% dos pacientes e a resolução das consolidações. ^33 , 34^ Outros achados de TC, mas com menor frequência, são: linhas subpleurais, broncogramas aéreos, linfonodomegalias, espessamento e derrame pleurais e derrame pericárdico. ^35 , 36^

### 4.3. Angiotomografia de Artérias Coronárias

A ATAC pode ser utilizada nos pacientes com COVID-19 que evoluem com níveis elevados de troponina para exclusão de DAC. Nessa situação, a ATAC pode ser de grande ajuda para excluir ou confirmar uma síndrome coronariana aguda se o quadro clínico for incerto, substituindo a angiografia coronária invasiva e a exposição a ela associada de todos os membros da equipe do laboratório de cateterismo cardíaco. Outro papel importante e emergente da ATAC na pandemia é como substituta da ETE para descartar trombo no apêndice atrial esquerdo antes da cardioversão elétrica, limitando a exposição do ecocardiografista. ^16^

A Sociedade de Tomografia Computadorizada Cardiovascular lançou um guia de recomendações para auxiliar os médicos quando da realização dos exames de ATAC durante a pandemia, tendo em vista a necessidade de priorizar os exames urgentes e a TC de tórax nos pacientes com COVID-19. ^37^ Nas seguintes situações, os exames são considerados urgentes e devem ser realizados de 1 hora até 4 semanas: ^37^

- Dor torácica aguda com suspeita de DAC;
- DAC estável em pacientes de risco para eventos ou preocupação de anatomia coronariana;
- Paciente com necessidade urgente de correção de cardiopatia estrutural;
- Avaliação do apêndice atrial esquerdo em pacientes com fibrilação atrial aguda antes da reversão para ritmo sinusal;
- Avaliação de cardiomiopatia em paciente com probabilidade pré-teste baixa de DAC, em que a ATAC mudará a conduta;
- Avaliação de disfunção de dispositivos de assistência ventricular;
- Disfunção sintomática de prótese valvar, endocardite com acometimento perivalvar e suspeita de abscesso;
- Novo tumor cardíaco com suspeita de etiologia maligna;
- Necessidade de descartar trombo intracavitário.

**Figura 2:**
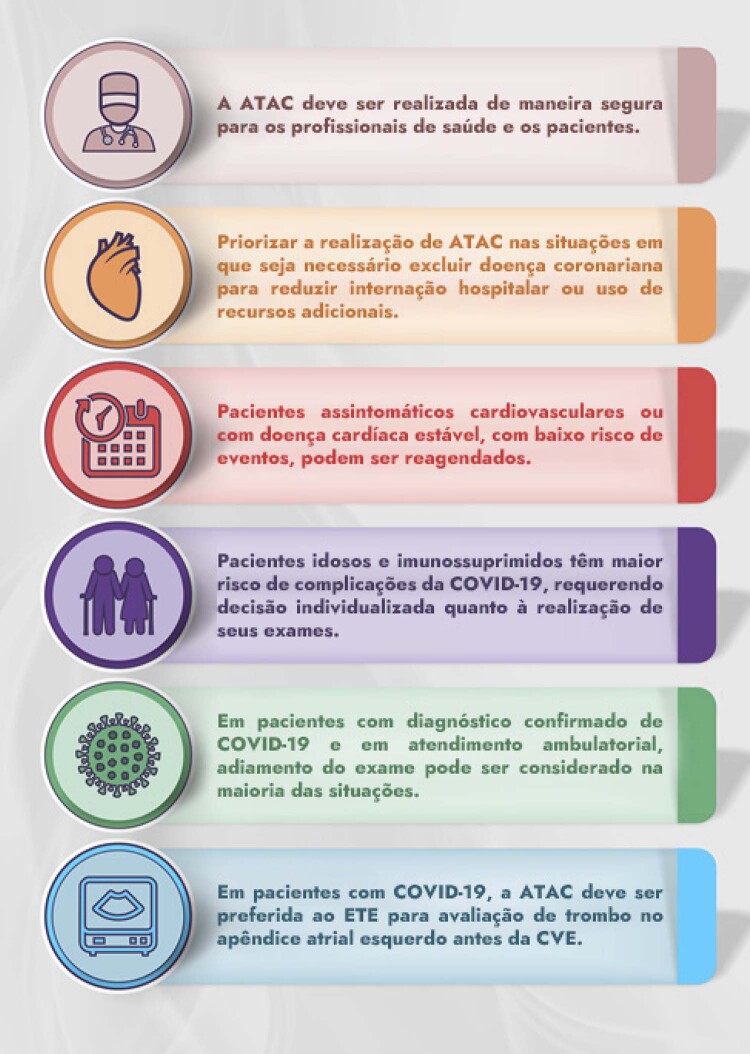
**–** Recomendações da realização de exames de angiotomografia de artérias coronárias durante a pandemia da COVID-19. ATAC: angiotomografia de artérias coronárias; COVID-19: doença do coronavírus 2019; CVE: cardioversão elétrica; ETE: ecocardiograma transesofágico

Os procedimentos ambulatoriais considerados eletivos podem ser reagendados para momento oportuno, entre 4 e 8 semanas. ^37^ Entretanto, é recomendável que se tenha cautela na avaliação de quais procedimentos podem ser adiados. Pode-se considerar o uso da telemedicina no auxílio à decisão e avaliação dos critérios. A Figura 2 sumariza as recomendações que podem auxiliar na decisão de realização ou adiamento dos exames.

A ATAC deve ser considerada em pacientes estáveis, sendo em geral necessária a administração de fármacos para controle da frequência cardíaca e vasodilatores coronarianos. Idealmente, deve-se optar por protocolos com baixa dose de radiação e de contraste. ^38^ Importante observar e descrever no laudo dos exames os achados pulmonares que possam auxiliar no manejo clínico do paciente.

### 4.4. Ressonância Magnética Cardíaca

A RMC pode ter importância na investigação etiológica de disfunção ventricular nova observada nos pacientes com COVID-19. Pacientes com troponinas positivas, disfunção miocárdica e arritmia grave/alterações eletrocardiográficas não explicadas por outros métodos podem ser candidatos à realização de RMC.16 Miocardite e síndrome de Takotsubo são etiologias sugeridas da disfunção ventricular relacionada ao SARS-CoV-2. ^23 , 25 , 39 , 40^

O diagnóstico de miocardite segue o mesmo critério das outras etiologias, habitualmente utilizando-se os critérios diagnósticos de Lake Louise, nos quais observam-se a presença de disfunção ventricular global ou segmentar, edema miocárdico, pericardite e/ou realce tardio de padrão não isquêmico. ^41 - 43^ A função ventricular esquerda pode estar preservada em alguns pacientes com miocardite. ^43^ O protocolo recomendado deve ser o mais curto possível, objetivando responder às perguntas do clínico. ^44^

**Figura 3:**
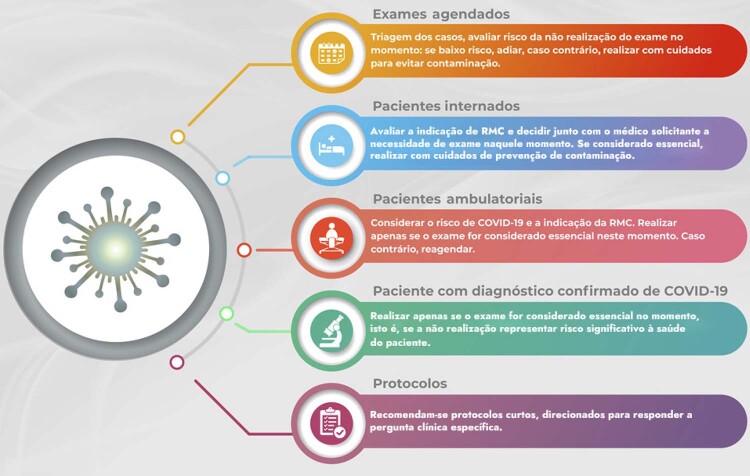
– *Recomendações de realização de exames de ressonância magnética cardíaca durante a pandemia de COVID-19. COVID-19: doença do coronavírus 2019; RMC: ressonância magnética cardíaca.*

Quanto aos exames eletivos, seu adiamento pode ser considerado se o médico requisitante julgar que não gera risco adicional à saúde do paciente. É necessário que o médico responsável pela realização do exame entre em contato com o solicitante para que a decisão de adiamento seja a opção mais segura para o paciente ( Figura 3 ). Em se decidindo pela realização do exame, o menor número de profissionais deve estar em contato com o paciente. Medidas de precaução devem ser tomadas durante todo o exame e o transporte. ^16^ Os profissionais devem ser treinados para o uso correto de EPI. Se possível, deve haver apenas um aparelho dedicado aos pacientes com COVID-19 suspeita ou confirmada. A limpeza do aparelho e da sala deve ser feita após a realização dos exames. ^45^

## 5. Procedimentos Intervencionistas

A pandemia pela COVID-19 representa um desgaste sem precedentes nos sistemas de saúde em todo o mundo, mais do que nunca exigindo eficiência na utilização de recursos e aumentando a busca por assistência justa, consistente, ética e eficiente. A decisão de realizar um procedimento de cardiologia intervencionista durante uma pandemia precisa levar em consideração os riscos de exposição viral da equipe de saúde, a utilização desnecessária de recursos e o potencial benefício do método.

### 5.1. Recursos Humanos

Recomendamos que cada unidade tome as medidas apropriadas para separar trabalhadores em grupos para que possíveis quarentenas possam ser aplicadas a grupos dentro de cada unidade e não à unidade como um todo. Idosos (idade > 65 anos), pessoas com doença cardíaca ou pulmonar crônica, DM ou HAS apresentam maior risco de doença grave após a COVID-19. Portanto, pode ser desejável minimizar a exposição direta da equipe de saúde com essas características a casos presumidos ou confirmados de COVID-19.

### 5.2. Indicação de Procedimentos

É importante uma avaliação cuidadosa da urgência clínica de um procedimento intervencionista durante o período de pandemia. Idealmente essa deve ser uma decisão conjunta entre o médico que vai realizar o procedimento, o cardiologista clínico e o paciente.

### 5.3. Doença Arterial Coronariana Estável

A avaliação do perfil de risco deve ser individualizada, considerando características clínicas, exames complementares e sintomatologia. De forma geral, a recomendação é que procedimentos eletivos para DAC estável devam ser adiados durante a pandemia. Pacientes com DAC estável, como os avaliados no estudo ISCHEMIA, possuem evolução favorável com tratamento clínico otimizado. Vale ressaltar que, no estudo ISCHEMIA, não foram incluídos pacientes com taxa de filtração glomerular estimada abaixo de 30ml/min/1,73m ^2^ de área de superfície corporal, síndrome coronariana aguda recente, estenose de tronco de coronária esquerda desprotegido de pelo menos 50%, fração de ejeção do ventrículo esquerdo inferior a 35%, insuficiência cardíaca de classe funcional III/IV da NYHA e angina inaceitável, apesar do uso de terapia médica otimizada. ^46^ No estudo ISCHEMIA, ficou demonstrada uma maior incidência de infarto agudo do miocárdio nos pacientes com DAC estável que ficaram em tratamento conservador quando comparados aos pacientes submetidos a revascularização. No entanto, isso só ocorreu após o sexto mês de seguimento, corroborando o adiamento de procedimentos intervencionistas nesse subgrupo de pacientes. ^46^

### 5.4. Síndrome Coronariana Aguda sem Supradesnivelamento de ST (SCASSST)

Primeiramente é importante ressaltar que 7-22% dos pacientes com COVID-19 apresentam quadro de injúria miocárdica com grande elevação de marcadores de necrose miocárdica, podendo corresponder a infarto agudo do miocárdio tipo 2 ou miocardite. ^7 , 10^ Deve-se tentar diferenciar infarto tipo 2 de síndrome coronariana aguda “primária”, com consideração de adiamento da estratificação invasiva no primeiro, principalmente se o paciente estiver hemodinamicamente estável.

**Figura 4:**
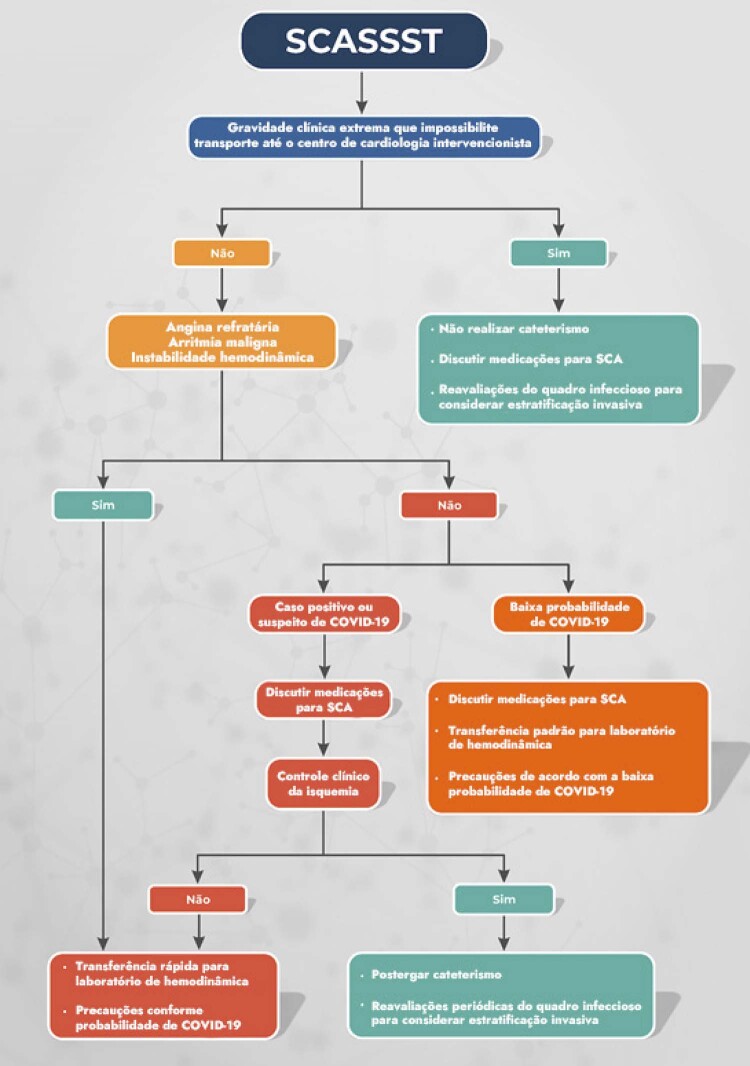
– *Fluxograma proposto para tratamento de síndromes coronarianas agudas sem supradesnivelamento do segmento ST. COVID-19: doença do coronavírus 2019; SCASSST: síndrome coronariana aguda sem supradesnivelamento do segmento ST; SCA: síndrome coronariana aguda.*

Para a maioria dos pacientes com SCASSST e suspeita de COVID-19, será possível realizar testes de diagnóstico para COVID-19 antes do cateterismo cardíaco e permitir uma decisão mais consciente sobre o controle de infecção. Pacientes com SCASSST instáveis, cuja instabilidade se deva à síndrome coronariana aguda, devem seguir fluxo de atendimento de urgência. A Figura 4 propõe um fluxograma para atendimento de casos confirmados de SCASSST de acordo com o diagnóstico de COVID-19.

Agilidade na alta após revascularização provavelmente será importante em termos de maximização da disponibilidade de leitos hospitalares e redução da exposição do paciente dentro do hospital. Acompanhamento por telemedicina pode ser uma ferramenta adicional neste período em que se recomenda minimizar o deslocamento de pessoas. ^47^

### 5.5. Infarto Agudo do Miocárdio com Supradesnível de ST (IAMCSST)

O IAMCSST é uma patologia de alta morbimortalidade e intervenção coronária percutânea primária (ICPP) deve ser considerada a terapia de escolha. ^48^ No entanto, diante da atual sobrecarga imposta aos sistemas de saúde pela COVID-19, alguns centros têm recomendado a fibrinólise como primeira linha de tratamento do IAMCSST. ^49^ Esse é um tema controverso, que deve levar em consideração a probabilidade de diagnóstico para COVID-19, a gravidade clínica do paciente, a disponibilidade de recursos e o tempo estimado para realização da ICPP.

**Figura 5:**
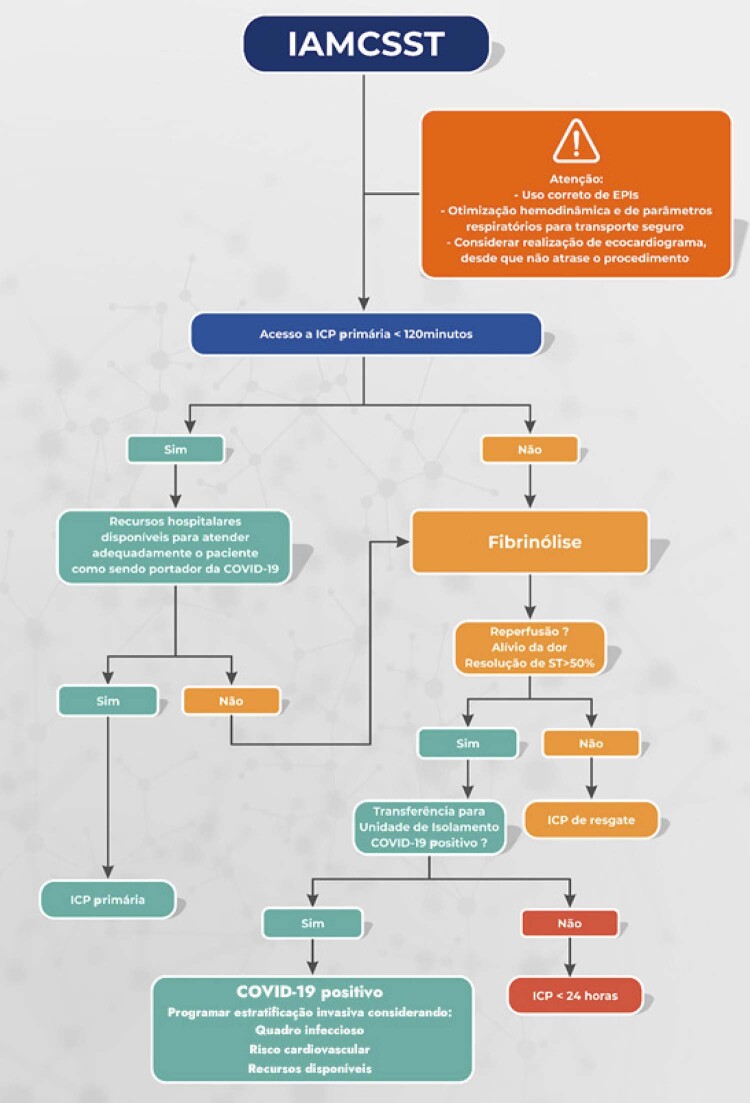
– *Fluxograma do atendimento de infarto agudo do miocárdio com supradesnivelamento do segmento ST (IAMCSST). Além da estratificação de risco cardiovascular, considerar agilidade para reperfusão, tempo de isquemia, recursos disponíveis para atendimento adequado do paciente. Pela necessidade de atendimento emergencial, todo paciente com IAMCSST deve ser considerado como potencial portador de COVID-19 e tratado de acordo com as medidas de isolamento adequadas até investigação do quadro infeccioso. EPIs: equipamentos de proteção individual; ICP: intervenção coronariana percutânea.*

No momento da confecção deste manuscrito, recomendamos que a ICPP deva ser o tratamento de escolha para IAMCSST em portadores de COVID-19. Se os recursos ficarem limitados, a gravidade clínica do paciente dificulte o transporte para o laboratório de cateterismo e o tempo porta-balão for inadequado, a equipe de cardiologia poderá decidir usar trombolíticos para pacientes com COVID-19 e IAMCSST em vez de ICPP. Em centros hospitalares sem acesso a laboratório de hemodinâmica, a fibrinólise mantém-se como tratamento padrão. ^50^ A Figura 5 propõe um algoritmo que leva em consideração o atendimento ao IAMCSST no cenário atual de pandemia. Pela necessidade de atendimento emergencial do IAMCSST, sugerimos que todo paciente com IAMCSST seja considerado inicialmente como portador de COVID-19, priorizando-se o quadro cardiovascular até que a investigação da infecção possa ser feita de forma adequada.

Vale ressaltar que pacientes com COVID-19 podem cursar com elevações do segmento ST, difusas ou localizadas, sem a presença de lesão obstrutiva que justifique a alteração. ^51^ Aqueles com DAC obstrutiva apresentavam níveis mais elevados de troponina e dímero-D. ^51^ Dessa forma, recomenda-se cautela na interpretação dos achados eletrocardiográficos, especialmente em pacientes com quadro pulmonar grave, para os quais as condições de transporte não sejam seguras. A realização de ecocardiograma, desde que não atrase a realização da ATAC quando indicada, pode ser considerada nesse cenário. ^51^

### 5.6. Procedimentos para Tratamento de Cardiopatia Estrutural Durante a Pandemia:

- **Troca valvar aórtica por cateter (TAVI):** A estenose aórtica (EA) é uma doença progressiva, que acomete um grupo de pacientes que pode ser muito idoso e estar já vulnerável à morte por infecções. A importância e a urgência clínica da TAVI exigiriam tomada de decisão conjunta pela equipe multidisciplinar envolvida (clínico, intervencionista, cirurgião). Essa decisão deve pesar o risco de expor o paciente à contaminação por COVID-19 por estar fora de seu ambiente domiciliar e o risco de um evento agudo, potencialmente fatal. ^52^ Encorajamos o acompanhamento próximo de pacientes com indicação de TAVI, com utilização de telemedicina durante o período de pandemia. Pacientes com EA importante e que se encontram assintomáticos podem ser considerados para acompanhamento ambulatorial. Aqueles com fatores complicadores à ecocardiografia (Vmax > 5,0 m/s, área valvar < 0,7 cm ^2^ , gradiente médio ventrículo esquerdo/aorta > 60 mmHg), pacientes com quadro de síncope, com redução de fração de ejeção do ventrículo esquerdo pela EA e em classe funcional III/IV (NYHA), que são considerados de maior risco para eventos, ^53^ idealmente não devem ter a TAVI adiada. Comparada à cirurgia aberta de troca valvar aórtica, a TAVI pode reduzir a demanda por unidade de terapia intensiva e por serviços anestésicos durante uma pandemia. Se a TAVI for realizada, a triagem pré-procedimento com PCR para COVID-19 pode ajudar na redução do risco da equipe.
- **Clipe mitral:** pacientes instáveis podem ser considerados para o clipe mitral se os recursos permitirem. Para aqueles de menor risco, o procedimento deve ser adiado.
- **Fechamentos de forame oval patente e defeito do septo atrial:** recomenda-se o adiamento.
- **Fechamento de apêndice atrial esquerdo:** recomenda-se o adiamento.
- **Outros procedimentos:** recomenda-se o adiamento, a menos que haja necessidade urgente de internação.

### 5.7. Redução da Propagação da Infecção

**5.7.1. Redução da propagação de gotículas:** Isso envolve medidas como o uso de máscaras cirúrgicas por pacientes com COVID-19 presumida ou confirmada. Todos os equipamentos não essenciais ao procedimento devem ser removidos da sala ou cobertos antes de o paciente entrar. Além disso, reduzir o número de pessoas que circulam na sala de procedimentos durante cada caso será importante para minimizar a exposição e a transmissão da infecção.50 A limpeza profunda e a desinfecção meticulosa após os procedimentos de cateterismo em pacientes com COVID-19 são importantes para o controle da infecção. A desinfecção por luz ultravioleta também pode ser uma estratégia razoável a ser empregada. Procedimentos de limpeza completos podem exigir tempo extra; portanto, se possível, esses casos devem ser o último procedimento do dia. Quando possível, realizar os procedimentos à beira do leito (marca-passo provisório, balão intra-aórtico) em pacientes com COVID-19 suspeita ou confirmada, visando a minimizar a necessidade de remoção do paciente de uma sala de isolamento e evitar o risco de exposição adicional através do transporte para o laboratório de hemodinâmica. ^50^

**5.7.2. Pacientes que necessitam de intubação, aspiração ou reanimação cardiopulmonar:** Intubação, sucção e reanimação cardiopulmonar ativa podem resultar em aerossolização de secreções respiratórias, aumentando a probabilidade de exposição pessoal. ^54^ Os pacientes que já estão intubados representam menos risco de transmissão para a equipe já que sua ventilação é gerenciada através de circuito fechado. ^55^ Pacientes com COVID-19 suspeita ou confirmada e que requerem intubação devem ser intubados antes da chegada ao laboratório de cateterismo. Além disso, pode ser necessário diminuir o limiar para se considerar a intubação em um paciente com problemas respiratórios limítrofes para evitar intubação de emergência na sala de cateterismo. ^55^ A cooperação com as equipes de cuidados intensivos e anestesia no gerenciamento das vias aéreas será fundamental para evitar a propagação da infecção.

### 5.8.Laboratório de Hemodinâmica Dedicado

Uma sala dedicada para atendimento de casos suspeitos/positivos objetiva reduzir o risco de infecção para os profissionais de saúde e minimizar a contaminação por vírus de outras salas. Se houver mais de uma sala de procedimento disponível, deve-se ter uma dedicada à COVID-19 e outra para os procedimentos “limpos”. Isso não garante que o “laboratório limpo” não seja contaminado a qualquer momento, mas serve para minimizar os riscos e otimizar o fluxo do paciente (especialmente para aqueles com “baixo risco de exposição”) no laboratório de hemodinâmica. É recomendável explorar com a engenharia do hospital se as salas de procedimento podem ter “pressão negativa de ar”. O entendimento do sistema de ar condicionado é importante, devido à possibilidade de expor outras partes do hospital à contaminação viral com um único procedimento. ^50^

**5.8.1. Medidas de controle administrativo** : Deve-se restringir a entrada de fornecedores, visitantes, observadores, coordenadores de pesquisa e qualquer pessoal não essencial ao laboratório de hemodinâmica na medida do possível durante a pandemia. ^55^

**5.8.2. Abordagem ao Paciente:** É importante realizar uma avaliação de risco de infeção por SARS-CoV-2 antes de submeter o paciente ao procedimento intervencionista. Recomenda-se organização para minimizar os tempos de espera nas áreas comuns do hospital antes ou depois do procedimento. ^7^ Todos os pacientes devem ser interrogados quanto a sintomas respiratórios, febre ou contato próximo com casos suspeitos/positivos antes de entrar na sala; também é recomendável medir a temperatura de todos os pacientes. ^55^

- **Abordagem do paciente sem confirmação de infecção por SAR-CoV-2:** Dada a situação atual e a possibilidade de tratar pacientes assintomáticos ou não diagnosticados, recomendamos medidas de proteção meticulosas. Os pacientes deverão utilizar máscara cirúrgica antes de entrar na sala. O cardiologista intervencionista deve tomar medidas de segurança que incluem a higienização adequada das mãos e o uso de avental estéril e impermeável, luvas estéreis, óculos, touca que cubra os cabelos e máscara cirúrgica. Auxiliares, enfermeiras e circulantes devem utilizar óculos, luvas, touca e máscara cirúrgica. ^55^
- **Abordagem do paciente com suspeita ou confirmação de infecção por SARS-CoV-2:** Os procedimentos que envolvam vias aéreas e/ou manipulação esofágica devem ser considerados como de alto risco. Somente pessoal essencial deve entrar na sala e as portas permanecerão fechadas o tempo todo. Evite sair da sala com equipamento contaminado (avental, luvas, máscara) para pegar material ( *stents* , cateteres). Idealmente o material utilizado para o procedimento ficará fora de sala. Um circulante ficará somente fora da sala e entregará exclusivamente o material necessário para o procedimento para outro circulante que ficará somente dentro da sala. Os medicamentos devem ser preparados antes que o paciente entre na sala.

O paciente usará máscara cirúrgica, que atua como uma barreira às secreções. O responsável pela transferência de um paciente com COVID-19 da maca para a mesa da sala de procedimentos deve usar EPI, incluindo bata impermeável a fluidos, touca, luvas que cobrem o pulso, óculos de proteção e máscara FFP2/N95. ^56^ No final da transferência, deve tirar a roupa conforme indicado abaixo, lembrando-se de nunca remover a máscara enquanto estiver dentro da sala. ^55^

**5.8.3. Paramentação:** O cardiologista intervencionista deve lavar as mãos, usar avental impermeável reforçado (se não for impermeável, é necessário adicionar um avental de plástico), luvas duplas, óculos plumbíferos ou óculos convencionais, barreira de proteção facial (escudo facial ou face *shield* ) e máscara de filtro de alta eficiência do tipo FFP2/N95. ^56^ Os auxiliares, enfermeiros e circulantes devem utilizar luvas, touca, bata impermeável e máscara FFP2/N95. Uma máscara cirúrgica deve ser colocada sobre a máscara FFP2/N95. É recomendável usar sapatos fechados. ^56^

**Figura 6:**
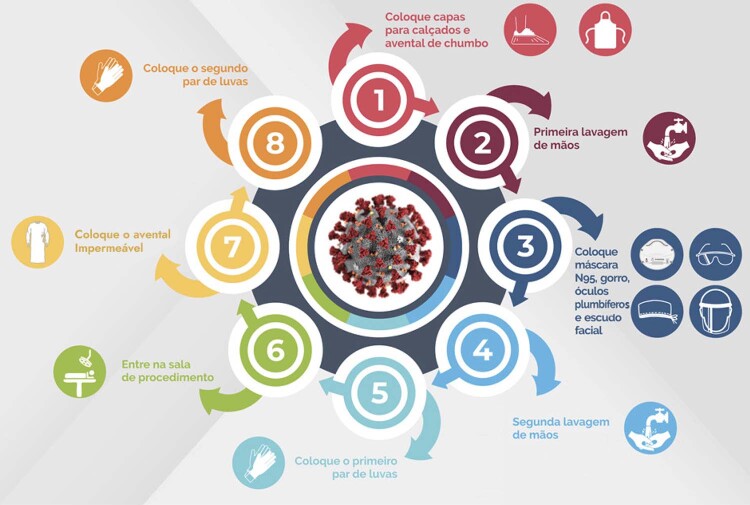
– *Procedimentos para paramentação do cardiologista intervencionista em casos suspeitos/confirmados de COVID-19.*

**5.8.3.1. Passo a passo para a paramentação em casos de COVID-19 suspeita ou confirmada** ( Figura 6 )

**Fora de Sala**

- Certifique-se de não usar joias ou adereços
- Recolha o cabelo (se necessário)
- Coloque o avental de chumbo e o propé
- Realize uma higiene adequada das mãos
- Coloque a máscara FFP2/N95. As ligas devem estar nos seguintes pontos: a inferior, na parte superior do pescoço; e a superior, no topo da cabeça. Em seguida, deve-se ajustar ao nível da ponte nasal e das bochechas, para que se isole e não haja vazamentos.
- Coloque a touca
- Coloque a máscara cirúrgica acima da N95
- Coloque os óculos e o escudo facial
- Realize desinfecção das mãos com álcool gel ou espuma
- Coloque o primeiro par de luvas
- Entre em sala

Dentro de Sala

- Coloque o avental impermeável
- Coloque o segundo par de luvas

**Figura 7:**
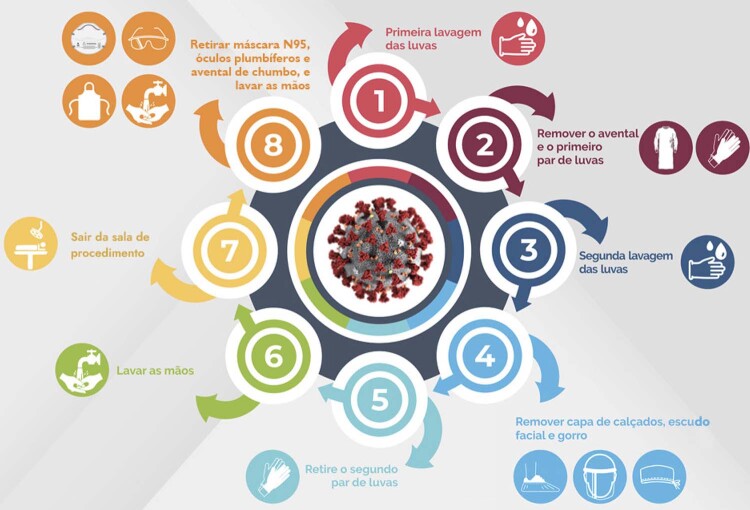
– *Procedimentos para o cardiologista intervencionista se desparamentar em casos suspeitos/confirmados de COVID-19.*

**5.8.3.2. Passo a passo para se desparamentar em casos de COVID-19 suspeita ou confirmada** ( Figura 7 )

**Dentro de Sala**

- Desinfete as luvas (externas) com álcool gel ou espuma
- Remova o avental e, simultaneamente, o par de luvas externas e jogue-as no recipiente de risco biológico-infectante (não empurre o avental para dentro do recipiente para evitar o aerossol, pois ele pode estar infectado)
- Desinfete as luvas (internas) com álcool gel ou espuma
- Retire o gorro, o escudo facial e o propé
- Retire o segundo par de luvas (internas)
- Desinfete as mãos com álcool gel ou espuma
- Saia de sala

### Limitações do Presente Documento

Deve-se reconhecer que este documento está sendo escrito em um momento em que não há ainda um entendimento completo da transmissão, gravidade e estratégia de tratamento apropriada para a COVID-19. As sugestões neste documento são baseadas em evidências limitadas e as recomendações estão sujeitas a mudanças.

## Conclusões

O crescimento exponencial do número de pacientes infectados tem gerado enorme sobrecarga nos serviços de saúde, com necessidade de implementação urgente de medidas que possam conter a infecção e restringir sua disseminação. Os pacientes com COVID-19 apresentam complicações cardiovasculares, necessitando, muitas vezes, de procedimentos diagnósticos de imagem para auxílio no seu manejo. A identificação correta dos pacientes que necessitam de exames de imagem e de procedimentos intervencionistas deve ser criteriosa, cautelosa e ética, priorizando a saúde do paciente e o uso racional de recursos.
