# Supplementary material for: Imagem Cardiovascular e Procedimentos Intervencionistas em Pacientes com Infecção pelo Novo Coronavírus
Source: Arq Bras Cardiol. 2020 Jul 28;115(1):111–26. [Article in Portuguese] doi: 10.36660/abc.20200370 (PMC8384321; doi:10.36660/abc.20200370)
Supplement: Translation

# Translation: Cardiovascular Imaging and Interventional Procedures in Patients with Novel Coronavirus Infection

**Authors:** Isabela Bispo Santos da Silva Costa, Carlos Eduardo Rochitte, Carlos M. Campos, Silvio Henrique Barberato, Gláucia Maria Moraes de Oliveira, Marcelo Antônio Cartaxo Queiroga Lopes, Cesar Higa Nomura, Alexandre A. Abizaid, Giovanni Cerri, Roberto Kalil, Ludhmila Abrahão Hajjar

**Affiliations:** 1 Brasil Universidade de São Paulo Instituto do Câncer do Estado de São Paulo, São Paulo, SP - Brasil; 2 Brasil Universidade de São Paulo Instituto do Coração, São Paulo, SP - Brasil; 3 Brasil Hospital do Coração, São Paulo, SP - Brasil; 4 Brasil Hospital Pró-Cardíaco, Rio de Janeiro, RJ - Brasil; 5 Brasil Universidade de São Paulo Instituto do Coração - Hemodinâmica e Cardiologia Intervencionista, São Paulo, SP - Brasil; 6 Brasil Hospital Israelita Albert Einstein - Hemodinâmica e Cardiologia Intervencionista, São Paulo, SP - Brasil; 7 Brasil CardioEco -Centro de Diagnóstico Cardiovascular, Curitiba, PR - Brasil; 8 Brasil Quanta Diagnóstico – Ecocardiografia, Curitiba, PR - Brasil; 9 Brasil Universidade Federal do Rio de Janeiro – Cardiologia, Rio de Janeiro, RJ - Brasil; 10 Brasil Hospital Alberto Urquiza Wanderley - Hemodinâmica e Cardiologia Intervencionista, João Pessoa, PB - Brasil; 11 Brasil Hospital Metropolitano Dom José Maria Pires, João Pessoa, PB - Brasil; 12 Brasil Sociedade Brasileira de Cardiologia, Rio de Janeiro, RJ - Brasil; 13 Brasil Hospital Sírio Libanês, São Paulo, SP - Brasil

**Keywords:** Coronavirus, COVID-19, Pandemics, Communicable Diseases, Emergency, Cardiovascular Diseases/prevention and control, Diagnostic Imaging, Medical Examination/methods, Diagnostic Techniques and Procedures

## Abstract

The coronavirus disease 2019 (COVID-19) pandemic is a huge challenge to the health system because of the exponential increase in the number of individuals affected. The rational use of resources and correct and judicious indication for imaging exams and interventional procedures are necessary, prioritizing patient, healthcare personnel, and environmental safety. This review was aimed at guiding health professionals in safely and effectively performing imaging exams and interventional procedures.

## 1. Introduction

Coronavirus disease 2019 (COVID-19), caused by severe acute respiratory syndrome coronavirus 2 (SARS-CoV-2), has become a huge challenge worldwide. The rapid spread of the infection has grown into a global level pandemic. As by May 20th, COVID-19 had already reached 185 countries, with 4,995,127infected individuals and 160,706 deaths.^1^ Despite underreporting due to test unavailability, Brazilian statistics have shown increasing numbers, with 291,579 infected patients and 18,859 deaths registered to date.^2^

The most effective strategy to control COVID-19 spread is home confinement, via quarantine and social distancing.^3^ Hospitals, clinics and medical offices have been following the recommendations of national and international medical societies to protect patients without COVID-19 from the risk of infection, while, at the same time, providing adequate care to those with COVID-19.^4 , 5^ In light of this, procedures considered elective should be timely rescheduled.^6^

The proper management of infected patients requires the adoption of a series of measures involving the interaction of several hospital sectors and the training of multidisciplinary teams. The majority of patients with the most severe COVID-19 forms have comorbidities, cardiovascular diseases being frequent.^7 - 9^ In addition, cardiovascular complications of COVID-19 occur in 7% to 40% of the cases, manifesting as myocardial injury, thrombosis, ventricular dysfunction, myocarditis, arrhythmias, and shock.^10 - 12^ These complications have significant prognostic implications, such as a high mortality rate.^11^

The diagnosis and follow-up of patients with those complications usually require performing imaging tests, such as electrocardiography, transthoracic echocardiography (TTE), computed tomography (CT), in addition to cardiac magnetic resonance imaging (CMRI) and coronary computed tomography angiography (CCTA). These tests should not be performed routinely in all infected patients, their indication being preferably based on the benefit added to the patient’s care and considering the safety of the staff conducting the tests. The need for rational, responsible, and thorough use of resources reinforces the importance of the clinician not only in identifying patients who need the test, but also in selecting the proper tests and in accurately interpreting their findings.

This review was aimed at: a) helping physicians to properly indicate and implement cardiovascular tests and interventional procedures in their clinical practice for patients with suspected or confirmed COVID-19; b) guiding physicians to safely perform the tests and procedures, preventing environmental and healthcare personnel contamination.

## 2. Approach to patients with suspected or confirmed COVID-19

The approach to patients with suspected or confirmed COVID-19 should begin with the proper characterization of their signs and symptoms. Those with mild symptoms, such as cough, fever, and sore throat, can be followed up in low-complexity units or at their homes. Those with more severe symptoms (oxygen saturation <94% in room air, respiratory distress, tachypnea, hypotension, acute respiratory failure) should be referred to specialized centers. On the initial contact, symptomatic individuals should be given a surgical mask and directed to a specific room aimed at respiratory isolation; in addition, they should receive instructions on hand hygiene to prevent contamination of the environment and other individuals.^4^

The identification of at-risk patients should comprise the assessment of clinical comorbidities known to be associated with a more severe course of the disease.^7 , 13^ Patients with arterial hypertension (AH), chronic cardiovascular disease, diabetes mellitus (DM), chronic obstructive pulmonary disease (COPD), or chronic kidney disease, in addition to immune suppressed or elderly patients, are more susceptible to develop complications, being considered a risk group.^4^

Patients with severe symptoms and/or of the risk group are prone to develop COVID-19-related cardiovascular complications.^8 , 9^ Zhou et al.,^9^ in a cohort with 191 patients, have reported high prevalence of AH (30%), DM (19%), coronary arterial disease (CAD - 8%), and COPD (3%).^9^ Of the 54 deceased patients (28%), 67% had a comorbidity, AH being identified in 48%, DM in 31%, and CAD in 24%. Advanced age was an independent predictor of mortality.9

Other important markers of severity in those patients are high serum levels of troponin, NT-proBNP, and D-dimer. Those with troponin elevation had more severe forms of COVID-19, with a higher incidence of acute respiratory distress syndrome (ARDS) and death.^11^ High troponin levels are accompanied by an elevation in markers of inflammation, thrombosis and cardiac dysfunction, patients with those characteristics being more likely to develop acute heart failure and shock.^8 , 9 , 11^

On admission, patients with clinical or laboratory findings suggestive of more severe disease should have their cardiovascular function evaluated via clinical assessment, measurement of biomarkers, and imaging tests.^12 , 14^ The most often performed imaging tests and interventional procedures are described in the following sections.

## 3. Echocardiography

Echocardiography has a well-established role in the diagnosis, prognostic assessment, and therapeutic guidance of several cardiovascular diseases. However, because it requires a close contact between examiner and patient, it poses a high risk of contamination. The pandemic has called for the urgent reorganization of echocardiography laboratories to minimize the exposure to COVID-19 and ensure the protection of patients and healthcare personnel.^5^ In light of this, the Brazilian Society of Cardiology Cardiovascular Imaging Department has issued a document to aid healthcare professionals during this pandemic.^5^

Echocardiography should not be performed routinely during the pandemic, especially in patients with confirmed COVID-19. However, echocardiography professionals will continue to be exposed in certain clinical scenarios in which that exam can play a decisive role in the differential diagnosis and clinical management of more severely ill patients. COVID-19 is known to cause severe cardiovascular manifestations; in addition, previous cardiovascular disease is common in patients with COVID-19, being associated with worse prognosis.^9 , 14 , 15^

### 3.1. General Precautions

The provision of patient care amidst the pandemic should meet the following safety recommendations to minimize the risks of healthcare personnel and patient exposure to COVID-19: (a) to define whether the test is deemed essential on the occasion; (b) to assess in advance the risk of contamination; (c) to respect the general hand hygiene and contact restriction rules; and (d) to observe the rational and strict use of proper personal protective equipment (PPE) according to the test type and contamination risk.^5^

When the likelihood of COVID-19 is low (low-risk areas and negative SARS-CoV-2 test), TTE in an asymptomatic patient requires the echocardiographer to perform thorough hand hygiene and wear gloves and surgical mask, and the patient to wear surgical mask as well, during the test. When the risk is moderate to high (symptomatic patients with suspected or confirmed COVID-19), the safety measures include hand hygiene and use of gloves, surgical mask (or N95 mask, when available), gown, hair covers and eye protection (goggles or face shield) by the examiner. The patient must wear a surgical mask. When transesophageal echocardiography (TEE), an aerosol-generating procedure, is necessary, the N95 mask or similar must be added to the aforementioned PPE, for respiratory protection. In addition, shoe covers, as well as a protective cover for the transducer, are recommended. For patients with suspected or confirmed COVID-19 on non-invasive or invasive mechanical ventilation, respiratory protection should also be adopted when performing a TEE.^16^ For inpatients, bedside echocardiography should be preferred, taking proper protective measures and reducing the number of individuals in the room to the lowest possible. The devices and transducers should be thoroughly cleaned and disinfected right after use according to the manufacturer’s specifications. Echocardiography laboratory staff with the following characteristics should be kept away: aged over 60 years, immunosuppressed, pregnant, and with chronic diseases.^5 , 17^

### 3.2. Indications for Echocardiography in Patients at Low Risk of COVID-19

During the pandemic, the indication for echocardiography in patients at low risk of COVID-19 should be based on the proper use of the exam; in addition, the exam should only be performed if the resulting information is deemed essential for the management of the case.^5^ All elective echocardiographies, such as, TTE, TEE, stress echocardiography (SE) and fetal echocardiography (FE), should be postponed until such time as the pandemic has waned. The need for urgent echocardiography on an outpatient setting should be assessed on a case-by-case basis; however, an urgent exam is the one whose result can prevent an adverse event or hospitalization within 2 to 4 weeks.^17^ In light of this, urgent echocardiography is recommended in the following situations: suspicion of new symptomatic heart disease [New York Heart Association (NYHA) functional class III/IV]; worsening of preexisting heart failure with severe symptoms (syncope, chest pain, NYHA functional class III/IV); cancer therapy with cardiotoxic drugs and suspected heart failure or previous reduction in ejection fraction; suspected severe symptomatic aortic stenosis; high pretest probability of infective endocarditis in a patient with valvular prosthesis and acute symptoms.^17^ Routine echocardiography for the follow-up of patients without severe symptoms or noneligible individuals for urgent clinical, surgical or invasive therapy should be deferred or canceled. For inpatients, the indications for urgent echocardiography are usually the same as before the pandemic.

### 3.3. Indications for Echocardiography in Patients with Suspected or Confirmed COVID-19

Echocardiography remains an essential imaging technique during the coronavirus pandemic. The considerations “in whom”, “how” and “where” to use it are fundamental to reduce the risks of contamination, and, at the same time, to ensure high-quality medical care. Some authors advocate the use of TTE in all patients with complicated COVID-19 (electrocardiographic changes, increased troponin levels, moderate to severe symptoms requiring hospitalization),^12 , 18^ specially in the presence of previous cardiovascular disease. Although there is no formal indication supported by solid scientific evidence, it is worth noting the importance of assessing cardiac function because of the potential simultaneous occurrence of previous and acute cardiovascular disease in patients with severe COVID-19.

Zhou et al.^9^ have reported heart failure in 23% of patients with COVID-19 and associated it with higher mortality (51.9% versus 11.7%).^9^ It is not clear whether that heart failure rate was due to aggravation of a previous ventricular dysfunction, new heart disease or both. Patients with previous ventricular dysfunction can develop severe heart failure decompensation in severe COVID-19, accompanied by hypotension and/or cardiogenic shock. Several possibilities have been suggested for acute myocardial injury, such as direct viral effect (myocarditis), hypoxic injury, toxic effect via “cytokine storm”, vasospasm, thrombosis, myocardial stunning due to stress cardiomyopathy, and hemodynamic instability.^19 - 21^ The possibility that SARS-CoV-2 causes myocarditis has been widely discussed. In a series of 150 patients with COVID-19, the retrospective analysis of 68 deaths has attributed 53% of them to respiratory failure, 7% to myocarditis with circulatory shock, 33% to a combination of both, and 5% to unknown causes.^15^ The authors have used clinical data to diagnose fulminant myocarditis, with no biopsy confirmation. Similarly, fulminant myocarditis has been reported in patients with and without fever, who had chest pain, ST-segment elevation with no coronary obstruction, and severe ventricular dysfunction, and who responded to salvage therapy with corticoid and immunoglobulins.^22 , 23^ Although in these two studies CMRI had shown findings compatible with myocarditis, there was no histological confirmation.^22 , 23^

The differential diagnosis with myocarditis and stress cardiomyopathy necessarily includes acute coronary syndromes, which have also been reported in patients with COVID-19.^24 , 25^ The intense inflammatory response and hemodynamic changes associated with severe COVID-19 might increase the risk of rupture of atherosclerotic plaques and/or thromboembolic phenomena in susceptible patients.^14^ Even for those with neither fever nor cough, who have typical cardiac manifestations, COVID-19 should be considered in the differential diagnosis during the pandemic, and echocardiography can aid clinical judgment.

Cardiac arrhythmias are common in inpatients with COVID-19, being described in 16.7% of the cases in a Chinese cohort with 138 patients.^7^ Echocardiography can be useful, especially for malignant ventricular arrhythmias, by diagnosing left ventricular dysfunction or preexisting structural heart disease.

Regarding severe pneumopathy and ARDS, association with pulmonary hypertension and right ventricular dysfunction should be assessed. Pericardial infusion has been reported as an exam finding associated with myocarditis (myopericarditis), usually without significant hemodynamic repercussion.^22 , 23^

In the following clinical scenarios, the indication for echocardiography in patients with COVID-19 seems defensible:^12 , 17 , 18 , 26^

- Suspected heart failure
- Enlarged heart on chest X-ray
- Clinically significant arrhythmias
- Chest pain with electrocardiographic changes and/or troponin elevation
- Hemodynamic instability and/or shock
- Suspected pulmonary hypertension and/or right ventricular dysfunction

For patients with severe COVID-19 admitted to the intensive care unit, bedside, and preferably point-of-care, echocardiography is recommended on admission and during the course of disease.^5 , 12^

### 3.4. Special Protocols During the Pandemic

**3.4.1.Transthoracic echocardiography:** the exam should have its length reduced to a minimum and be targeted at the suspected diagnosis. Because the risk of contamination increases as the duration of the exam lengthens, the use of focused echocardiography, rather than complete TTE, has been recommended.^5 , 17 , 26^ Nevertheless, unnecessary repetition of exams should be avoided, and, according to the complexity of the case, complete TTE might be required to meet clinical demand. Images should be stored aiming at performing the off-line measurements, and electrocardiographic monitoring can be dismissed. Ideally one exclusive echocardiography device should be dedicated to patients with COVID-19, and it should remain in the contaminated areas. Additional protective measures can be adopted, such as plastic film wrapping of the device and/or interposition of an acrylic (or plastic) barrier between the examiner and the patient. Portable or pocket ultrasound devices can be easily covered, transported, and disinfected; however, their diagnostic resources are limited (point-of-care). Echocardiographic contrast agents might be useful, and their use should be anticipated to prevent additional circulation in the exam room.^26^ It is worth noting that contrast agents should not be used for critical patients with circulatory instability and severe pulmonary impairment.

**3.4.2. Focused (point-of-care) echocardiography:** may be important for the care of critical patients during the COVID-19 pandemic. Although not equivalent to complete TTE, focused echocardiography can confirm or exclude a specific diagnosis, supporting therapeutic decisions.^27^ It can be performed by properly trained physicians already providing direct care to the patient in the intensive care unit, thus contributing to reduce the exposure of the echocardiographer. Portable or pocket ultrasound devices should be preferably used to facilitate access to bed and further disinfection.

**3.4.3. Transesophageal echocardiography:** there is special concern regarding TEE, because of the high risk of equipment and healthcare personnel contamination with droplets and aerosols. Thus the incremental value of TEE over TTE should be carefully assessed, and TEE should be avoided in most cases.^16^ Whenever possible, other alternatives should be considered, such as repeating TTE or using another imaging technique with less contact between examiner and patient, such as CT and CMRI. To perform urgent TEE in hospitalized patients, the examiner should use complete PPE for respiratory protection, in addition to protective cover for the transducer.

**3.4.4. Stress echocardiography:** exercise stress echocardiography can increase the risk of contamination via droplets and should, thus, be deferred (patients at low risk of COVID-19) or not performed (patients with suspected or confirmed COVID-19). When there is proper indication and deferral is not possible or recommended, pharmacological stress echocardiography should be preferred for patients at low risk of COVID-19 (patient with cancer waiting for surgery and with high pretest probability of obstructive CAD). In addition, during the pandemic, selected cases of chronic CAD could be investigated by use of CCTA.

**3.4.5. Fetal echocardiography:** proper indication for FE remains the same during the pandemic, which is pregnancy with high risk of fetal heart disease;^16^ the exam should be performed outside the hospital setting. At the moment, there is no indication for routine FE in mothers with suspected or confirmed COVID-19.

**3.4.6. Lung ultrasonography:** is an agile tool to assess lung involvement and to follow up the result of bedside therapeutic interventions. The following findings have been reported in COVID-19: thickening and irregularity of the pleural line with loss of continuity; several patterns of B lines (focal, multifocal, confluent); and lung consolidations (frequently subpleural).^28^ Although those abnormalities are unspecific and found in other types of pneumonia, they are valuable to track the evolution of COVID-19 pneumonia. Pleural effusions are not frequent, and A lines appear during the recovery phase.

## 4. X-ray, Computed Tomography and Cardiac Magnetic Resonance Imaging

Pulmonary and cardiovascular imaging tests play an important role in the accurate diagnosis of COVID-19 complications. Chest X-ray is the most used exam, but CT might be necessary.^16^ The CMRI might be required in patients with suspected myocarditis and/or Takotsubo syndrome.^16^ However, before indicating CT and CMRI, some considerations should be made. Such tests pose a significant risk of contamination for patients and health professionals, not only those related to patient’s transportation but also to direct contamination during the exam. The CMRI and CT should only be performed if the information resulting from the tests could help the patient’s clinical management. Those exams should be indicated for stable patients with a minimum transportation risk, if performed at a safe environment, with strict adherence to local safety rules, and with the use of PPE by the professionals involved in patient’s transportation and image acquisition.^16^

### 4.1. Chest X-ray

Chest X-ray is usually the first imaging test performed in patients with COVID-19 because of its low cost and ease of access, mainly in hospitalized patients who cannot be safely transported.^29^ Chest X-ray has low sensitivity. In addition, X-ray findings are not specific to COVID-19, because they can also be associated with the flu syndrome, such as consolidations (47%), low-density opacities (33%), and pleural effusion (3%).^30^ The imaging findings are predominantly peripheral, occurring most often within 10 to 12 days.^30^Figure 1 summarizes the major chest X-ray findings.

**Figure 1:** – *Chest X-ray (XR) and computed tomography (CT) in COVID-19.*

### 4.2. Chest Computed Tomography

Chest CT is a tool to support the diagnosis, while COVID-19 confirmation is based on viral reverse transcriptase polymerase chain reaction (RT-PCR) or serological tests. Performing screening CT for the identification of COVID-19 should not be encouraged.^29^ Asymptomatic or mildly symptomatic patients should not undergo CT; however, for mildly symptomatic patients with access to neither RT-PCR nor serological tests, the benefit of performing CT is uncertain. For severely symptomatic, hospitalized patients, who can be transported safely, such as using a mask, CT should be considered when complications are suspected (pulmonary thromboembolism, pleural effusion, and superimposed bacterial infection).^29^Figure 1 summarizes CT findings and recommendations for performing CT.

The protocol recommends the use of low radiation doses, preferably with no contrast medium administration, which should be reserved for specific indications, such as to discard pulmonary thromboembolism.^29^ In the first days after symptom onset, CT can be normal, which does not exclude COVID-19. The CT sensitivity and specificity reported for COVID-19 vary widely (60% to 98%, and 25% to 53%, respectively), probably because of the retrospective nature of the studies published, including the lack of strict criteria for imaging diagnosis and procedural differences for confirming infection.^31^

The CT findings depend on when infected patients are imaged. In the initial phase, 57-98% of the patients will present usually bilateral, peripheral and rounded ground-glass opacities.^32^ From 5% to 36% of the patients will have a ‘crazy paving’ pattern at disease peak (5 to 8 days after symptom onset). Consolidations are present in 2% to 64% of the patients, commonly in the elderly and those with the disease severe form. Later in the course of the disease, a reticular pattern is observed in 48% of the patients, as is the gradual resolution of the consolidations.^33 , 34^ Other CT findings, although less frequent, in COVID-19 are as follows: subpleural lines, air bronchograms, lymph node enlargement, pleural thickening and effusion, and pericardial effusion.^35 , 36^

### 4.3. Coronary Computed Tomography Angiography

CCTA can be performed in patients with COVID-19 and high troponin levels to exclude CAD. In that situation, CCTA can be extremely helpful to exclude or confirm acute coronary syndrome if the clinical findings are uncertain, replacing invasive coronary angiography and the exposure of all cardiac catheterization laboratory (CCL) staff that comes with it. Another important and emerging role of CCTA during the pandemic is to replace TEE in ruling out a thrombus in the left atrial appendage before electrical cardioversion, limiting the exposure of the echocardiographer.^16^

The Society of Cardiovascular Computed Tomography has offered guidance with recommendations to help physicians when performing CCTA during the pandemic, considering the need to prioritize urgent exams and chest CT in patients with COVID-19.^37^ These are urgent indications, in which scanning should be performed within hours to 4 weeks:^37^

- Acute chest pain with clinical suspicion for CAD;
- Stable CAD at high risk for events or when there is concern for high-risk coronary anatomy;
- Patient requiring urgent structural correction of heart disease;
- Assessment of left atrial appendage in patients with acute atrial fibrillation prior to restoration of sinus rhythm;
- Assessment of cardiomyopathy in low pretest probability of CAD, only if CCTA will change management;
- Assessment of ventricular assist device dysfunction;
- Symptomatic prosthetic heart valve dysfunction, endocarditis, perivalvular extension of endocarditis, and possible valve abscess;
- New cardiac tumor suspected to be malignant;
- Need to rule out intracavitary thrombus.

Outpatient procedures considered elective can be rescheduled timely, within 4-8 weeks.^37^ However, the decision about which procedures defer should be carefully made. Telemedicine can help in decision making and criteria assessment. Figure 2 summarizes the recommendations to support the decision about performing or deferring the exams.

**Figure 2:** – *Recommendations for performing coronary computed tomography angiography during the COVID-19 pandemic. CCTA, coronary computed tomography angiography; TEE, transesophageal echocardiography; ECV, electrical cardioversion.*

Stable patients should be considered for CCTA, which usually requires the administration of heart rate control drugs and coronary vasodilators. Ideally, low radiation and contrast dose protocols should be chosen.^38^ It is worth observing and describing in the exam report the pulmonary findings that might aid the patient’s clinical management.

### 4.4. Cardiac Magnetic Resonance Imaging

CMRI can be important the etiological investigation of new ventricular dysfunction in COVID-19. Patients with high troponin levels, myocardial dysfunction and severe arrhythmia/electrocardiographic changes not explained by use of other methods can be candidates to undergo CMRI.^16^ Myocarditis and Takotsubo syndrome are suggested etiologies of the SARS-CoV-2-related ventricular dysfunction.^23 , 25 , 39 , 40^

The diagnosis of myocarditis follows the same criterion of the other etiologies, usually using the Lake Louise diagnostic criteria, which comprise the presence of regional or global ventricular dysfunction, myocardial edema, pericarditis and/or non-ischemic delayed enhancement.^41 - 43^ Left ventricular function can be preserved in some patients with myocarditis.^43^ The protocol recommended should be the shortest possible, aimed at answering the clinician’s questions.^44^

Regarding an elective exam already scheduled, its deferral can be considered if the requesting physician understands that does not increase the risk to the patient. The physician in charge of the exam should decide together with the requesting one about the safety for the patient of postponing the exam ( Figure 3 ). If deciding upon performing the exam, the lowest number of professionals should be in contact with the patient. Safety measures should be taken during the entire procedure and patient’s transportation.^16^ Healthcare professionals should be well educated on the proper use of PPE. Whenever possible, one imaging device should be dedicated to patients with suspected or confirmed COVID-19. Device and room cleaning should be performed after the exam.^45^

**Figure 3:** – *Recommendations for performing cardiac magnetic resonance imaging (CMRI) during the COVID-19 pandemic.*

## 5. Interventional Procedures

The COVID-19 pandemic has imposed an unprecedented stress to healthcare systems worldwide. More than ever, the situation calls for extraordinary efficiency in the use of resources and increases the need for fair, consistent, ethical, and efficient healthcare provision. The decision about performing an interventional cardiology procedure amidst a pandemic should balance the risk of healthcare staff’s exposure to the virus, the unnecessary use of resources, and the potential benefit to the patient.

### 5.1. Human Resources

Each service should take proper measures to separate workers into groups, so that possible quarantines can be applied to groups inside each service and not to the entire service. The elderly (age > 65 years), individuals with chronic heart or pulmonary disease, DM or AH are at higher risk of severe disease after COVID-19. Thus, minimizing the direct exposure of healthcare personnel with those characteristics to cases of presumed or confirmed COVID-19 might be advisable.

### 5.2. Indication for a Procedure

Thorough assessment of the clinical urgency of an interventional procedure during the pandemic is essential. Ideally, that should be a joint decision of the physician performing the procedure, the clinical cardiologist, and the patient.

### 5.3. Stable Coronary Artery Disease

Risk profile assessment should be individualized, considering clinical findings, complementary tests and symptoms. Usually, elective procedures for stable CAD should be postponed until after the pandemic. Patients with stable CAD, as those assessed in the ISCHEMIA study, have a favorable outcome with optimized clinical treatment. It is worth noting that the ISCHEMIA trial has not included patients with the following characteristics: estimated glomerular filtration rate lower than 30mL/min/1.73m^2^of body surface area; recent acute coronary syndrome; unprotected left main coronary artery stenosis of at least 50%; left ventricular ejection fraction lower than 35%; NYHA functional class III/IV heart failure; and unacceptable angina despite the use of optimized medical therapy.^46^ The ISCHEMIA trial has shown a higher incidence of acute myocardial infarction in patients with stable CAD under conservative treatment as compared to those submitted to revascularization. That, however, has occurred only after a six-month follow-up, corroborating the deferral of interventional procedures in that subgroup of patients.^46^

### 5.4. Non-ST-elevation Acute Coronary Syndrome (NSTE-ACS)

It is worth noting that 7-22% of the patients with COVID-19 have myocardial injury with significant elevation in myocardial necrosis markers, which might correspond to type 2 acute myocardial infarction or myocarditis.^7 , 10^ Type 2 acute myocardial infarction should be distinguished from “primary” acute coronary syndrome, and deferral of invasive stratification considered in the former, mainly if the patient is hemodynamically stable.

For most patients with NSTE-ACS and suspected COVID-19, diagnostic tests for COVID-19 might be performed before cardiac catheterization, allowing to a more sensible decision-making about infection control. Unstable patients with NSTE-ACS, whose instability is due to acute coronary syndrome, should follow the urgent care flow. Figure 4 shows a flowchart for the care of confirmed cases of NSTE-ACS according to the diagnosis of COVID-19.

**Figure 4:** – *Flowchart for the treatment of non-ST-elevation acute coronary syndromes. NSTE-ACS, non-ST-elevation acute coronary syndrome; ACS, acute coronary syndrome.*

Readiness to discharge after revascularization might be important to maximize the availability of hospital beds and reduce the patient’s exposure inside the hospital. Follow-up via telemedicine can be an additional tool in a time when restriction to people circulation is recommended.^47^

### 5.5. ST-elevation Acute Myocardial Infarction (STEMI)

STEMI has high morbidity and mortality, and primary percutaneous coronary intervention (PPCI) should be deemed the therapy of choice.^48^ However, in face of the current burden imposed to health systems by COVID-19, some centers have recommended fibrinolysis as the first-line treatment of STEMI.^49^ This is a controversial issue that should take into account the COVID-19 diagnosis probability, the patient’s clinical severity, the availability of resources, and the estimated time to perform PPCI.

At the time this article was written, PPCI was recommended as the treatment of choice for STEMI in patients with COVID-19. If resources become scarce, the clinical severity hinders patient’s transportation to the CCL, and the door-balloon time is inadequate, the cardiology staff might decide to use thrombolytics, rather than PPCI, for patients with COVID-19 and STEMI. In hospitals with no access to a CCL, fibrinolysis remains the standard treatment.^50^Figure 5 presents an algorithm with the care for STEMI in the current pandemic scenario. Because of the need for emergency care, all patients with STEMI should be considered initially as having COVID-19, and the cardiovascular findings should be prioritized until the infection can be properly investigated.

**Figure 5:** – *Flowchart for the treatment of ST-elevation acute myocardial infarction (STEMI). In addition to cardiovascular risk stratification, consider readiness to perform reperfusion, ischemia time, and resources available for patient’s proper care. Because of the need for emergency care, all patients with STEMI should be considered as potentially having COVID-19 and treated according to the proper isolation measures until the infection can be thoroughly investigated. PCI, percutaneous coronary intervention.*

It is worth noting that patients with COVID-19 can have diffuse or regional ST-segment elevations, with no obstructive lesion justifying the alteration.^51^ Those with obstructive CAD have higher levels of troponin and D-dimer.^51^ Thus, caution is recommended in interpreting the electrocardiogram, mainly in patients with severe pulmonary findings, whose transportation conditions are not safe. In that scenario, echocardiography can be considered, as long as it does not delay CCTA, when indicated.^51^

### 5.6. Procedures for Structural Heart Disease Management During the COVID-19 Pandemic

- **Transaortic valve implantation (TAVI):** Aortic stenosis (AS) is a progressive disease that affects patients with advanced age vulnerable to death from infection. The importance and clinical urgency of TAVI require a joint decision-making by a multidisciplinary team (clinical and interventional cardiologists and surgeon). That decision should weigh the risk of patients’ exposure to COVID-19 contamination against their risk of an acute, potentially fatal event.^52^ Patients with indication for TAVI should be closely followed up by telemedicine during this pandemic. Asymptomatic patients with significant AS can be followed up on an outpatient basis. Those with complicating echocardiographic findings (Vmax > 5.0 m/s, valvular area < 0.7 cm^2^, mean left ventricle/aorta gradient > 60 mm Hg), syncope, reduced left ventricular ejection fraction due to AS and NYHA functional class III/IV, who are at higher risk for events,^53^ ideally should not have their TAVI postponed. Compared to open-chest surgery for aortic valve replacement, TAVI can reduce the need for intensive and anesthesia care during a pandemic. If TAVI is to be performed, preprocedural screening with PCR for COVID-19 might reduce the risk for the healthcare personnel.
- **Mitral valve clip:** mitral valve clip procedure can be considered for unstable patients if resources allow and should be postponed for lower-risk patients.
- **Closures of patent*foramen ovale*and atrial septal defect:** should be postponed.
- **Left atrial appendage closure:** should be postponed.
- **Other procedures:** should be postponed unless urgent hospitalization is required.

### 5.7. Reducing the Spread of COVID-19

**5.7.1. Reducing droplets spreading:** Involves measures such as the use of surgical mask by patients with suspected or confirmed COVID-19. All nonessential equipment should be moved out of the CCL procedure room or covered with clear drapes before the patient’s arrival to the room. In addition, it is worth emphasizing the importance of reducing circulation in the procedure room to minimize exposure and infection spread.^50^ Deep cleaning and thorough disinfection of the room after CCL procedures involving patients with COVID-19 are important to control the infection. In addition, disinfection with ultraviolet radiation can be used. Thorough cleaning might require an extra time; thus, if feasible, a procedure in a patient with COVID-19 should be performed as the final one of the day. Whenever possible, the patient with suspected or confirmed COVID-19 should undergo bedside procedures (transient pacemaker, intra-aortic balloon) aiming at minimizing the need for moving the patient out from an isolation room and preventing the risk of additional exposure via transportation to the CCL.^50^

**5.7.2. Patients requiring intubation, aspiration or cardiopulmonary resuscitation:** Intubation, aspiration and active cardiopulmonary resuscitation can generate aerosol particles from respiratory secretions, increasing the likelihood of personal exposure.^54^ Patients already intubated pose a lower contamination risk to healthcare personnel, because they are on closed-loop ventilation.^55^ For patients with suspected or confirmed COVID-19 who need orotracheal intubation, this intervention should be performed before arrival to the CCL. In addition, intubation should be considered as early as possible in borderline patients to avoid the need for an urgent intubation in the CCL.^55^ The cooperation of the intensive care and anesthesia staffs for airway management is fundamental to prevent the infection spread.

**5.8. Dedicated Catheterization Laboratory**

Having a dedicated room for the care of suspected/positive COVID-19 cases is aimed at reducing the risk of infection for health professionals and minimizing the viral contamination of other rooms. In CCLs with more than one procedure room, one should be dedicated to COVID-19 and another to ‘clean’ procedures. This is no guarantee that the ‘clean’ CCL will not be contaminated at any time but can minimize the risks and optimize the flow of patients in the CCL, mainly of those at “low risk for exposure”. It is advisable to consult with the hospital engineering about the possibility of having “negative air pressure” procedure rooms. Understanding the air conditioning system is important, because one single procedure might expose other hospital areas to viral contamination.^50^

**5.8.1. Measures of management control.** Suppliers, visitors, observers, research coordinators and any nonessential individual for the CCL operation should refrain from entering the CCL during the pandemic.^55^

**5.8.2. Approaching the patient.** It is worth noting the importance of assessing the risk of SARS-CoV-2 infection before submitting the patient to the interventional procedure. Organization is recommended to minimize the waiting times in the hospital common areas before and after the procedure.^7^ All patients should be asked about respiratory symptoms, fever or close contact with suspected/positive cases before entering the CCL room, in addition to undergoing temperature check.^55^

- **Approach to patients without confirmed SAR-CoV-2 infection:** Given the current situation and the likelihood of treating asymptomatic or undiagnosed patients, careful protective measures are recommended. Patients should wear surgical mask before arrival to the room. The interventional cardiologist should adopt safety measures that include proper hand hygiene and the use of sterile and water-resistant gown, sterile gloves, goggles, hair covers, and surgical mask. Technologists, nurses and circulating technicians should use goggles, gloves, hair covers, and surgical mask.^55^
- **Approach to suspected or confirmed COVID-19 patients:** Procedures involving airway and/or esophageal manipulation should be considered of high risk. Only essential personnel should be granted access to the CCL room, whose doors should remain closed all time. Avoid exiting the room with contaminated equipment (gown, gloves, mask) to get material (stents, catheters). Ideally the material used in the procedure should remain outside the room. A circulating technician will remain outside the room exclusively providing the material necessary for the procedure to another circulating technician remaining exclusively inside the room. Medications should be prepared before patient’s arrival to the room.

The patient should wear a surgical mask, which acts as a barrier to secretions. The staff responsible for moving the COVID-19 patient from the litter to the CCL table should wear PPE, including water-resistant gown, hair covers, gloves covering the wrists, eye protection, and FFP2/N95 mask.56 At the end of transfer, the PPE should be removed as indicated in the following topic, noting that the mask should never be removed inside the CCL room.^55^

**5.8.3. Putting on PPE:** The interventional cardiologist should perform hand hygiene with soap and water, wear a reinforced water-resistant gown (if not impermeable, a plastic gown needs to be added), two pairs of gloves, protective lead goggles or conventional eye glasses, face shield, and high-efficiency filter mask of the FFP2/N95 type.^56^ Technologists, nurses and circulating technicians should use gloves, hair cover, water-resistant gown and FFP2/N95 mask. A surgical mask should be put on over the FFP2/N95 mask. Closed shoes are recommended.^56^

**5.8.3.1.Steps for putting on PPE in cases of suspected or confirmed COVID-19** ( Figure 6 )

**Figure 6:** – *Steps for putting on the personal protective equipment in cases of suspected or confirmed COVID-19.*

**Outside the CCL procedure room**

- Remove any personal items
- Hold hair fully back
- Put on the lead gown and shoe covers
- Perform proper hand hygiene
- Put on the FFP2/N95 mask, securing ties or elastic bands at middle of head and neck, fit flexible band to nose bridge and snug to face and below chin, to ensure isolation and no leak.
- Put on hair cover
- Put on surgical mask over the N95 mask
- Put on goggles and face shield
- Perform hand disinfection with alcohol gel or foam
- Put on the first pair of gloves
- Enter the CCL procedure room

Inside the CCL procedure room

- Put on the water-proof gown
- Put on the second pair of gloves

**5.8.3.2. Steps for removing PPE in cases of suspected or confirmed COVID-19** ( Figure 7 )

**Figure 7:** – *Steps for removing the personal protective equipment in cases of suspected or confirmed COVID-19.*

**Inside the CCL procedure room**

- Disinfect the external pair of gloves with alcohol gel or foam
- Remove the gown and simultaneously the external pair of gloves, discarding them in a waste container marked with the biological hazard symbol (do not push to avoid generating aerosol particles, because they can be infected)
- Disinfect the internal gloves with alcohol gel or foam
- Remove hair cover, face shield and shoe covers
- Remove the internal pair of gloves
- Perform hand disinfection with alcohol gel or foam
- Exit the procedure room

### Limitations of the present document

It is worth noting that this document is being written in a time when we do not completely understand COVID-19 transmission, severity, and proper treatment. The strategies herein suggested are based on limited evidence and recommendations might be subject to change.

## Conclusions

The exponential growth in the number of patients with COVID-19 has been a huge burden on health services, requiring the urgent adoption of measures that can contain the virus and restrain its spread. Patients with COVID-19 have cardiovascular complications and often require diagnostic imaging tests and procedures to support their management. The correct identification of patients who need imaging tests and interventional procedures should be judicious, careful, and ethical, prioritizing the patient’s health and the rational use of resources.
